# Oral Cancer Discrimination and Novel Oral Epithelial Dysplasia Stratification Using FTIR Imaging and Machine Learning

**DOI:** 10.3390/diagnostics11112133

**Published:** 2021-11-17

**Authors:** Rong Wang, Aparna Naidu, Yong Wang

**Affiliations:** 1 School of Dentistry, University of Missouri-Kansas City, Kansas City, MO 64108, USA; wangrong@umkc.edu; 2 Oral Surgery and Pathology, Truman Medical Center, Kansas City, MO 64108, USA; aparna.naidu@tmcmed.org

**Keywords:** Fourier transform infrared spectroscopy, FTIR imaging, spectral biomarker, multivariate analysis, machine learning, discriminant model, oral squamous cell carcinoma, oral epithelial dysplasia, oral potentially malignant disorder, risk stratification, early oral cancer detection

## Abstract

The Fourier transform infrared (FTIR) imaging technique was used in a transmission model for the evaluation of twelve oral hyperkeratosis (HK), eleven oral epithelial dysplasia (OED), and eleven oral squamous cell carcinoma (OSCC) biopsy samples in the fingerprint region of 1800–950 cm^−1^. A series of 100 µm × 100 µm FTIR imaging areas were defined in each sample section in reference to the hematoxylin and eosin staining image of an adjacent section of the same sample. After outlier removal, signal preprocessing, and cluster analysis, a representative spectrum was generated for only the epithelial tissue in each area. Two representative spectra were selected from each sample to reflect intra-sample heterogeneity, which resulted in a total of 68 representative spectra from 34 samples for further analysis. Exploratory analyses using Principal component analysis and hierarchical cluster analysis showed good separation between the HK and OSCC spectra and overlaps of OED spectra with either HK or OSCC spectra. Three machine learning discriminant models based on partial least squares discriminant analysis (PLSDA), support vector machines discriminant analysis (SVMDA), and extreme gradient boosting discriminant analysis (XGBDA) were trained using 46 representative spectra from 12 HK and 11 OSCC samples. The PLSDA model achieved 100% sensitivity and 100% specificity, while both SVM and XGBDA models generated 95% sensitivity and 96% specificity, respectively. The PLSDA discriminant model was further used to classify the 11 OED samples into HK-grade (6), OSCC-grade (4), or borderline case (1) based on their FTIR spectral similarity to either HK or OSCC cases, providing a potential risk stratification strategy for the precancerous OED samples. The results of the current study support the application of the FTIR-machine learning technique in early oral cancer detection.

## 1. Introduction

Oral cancer refers to a subgroup of head and neck malignancies that affect the lips, tongue, salivary glands, gingiva, floor of the mouth, buccal surfaces, and other intra-oral locations. It is one of the most prevalent cancers worldwide, with especially high incidence in low- and middle-income countries. Despite easy access to the oral cavity and new management strategies, oral cancer is still characterized by high morbidity and low survival rates, which are partially due to late diagnosis [1]. More than 90% of oral cancers are oral squamous cell carcinoma (OSCC), which are a heterogeneous group of cancers arising from the mucosal lining of the oral cavity. Most oral cancer cases are associated with lifestyle habits including smoking, smokeless tobacco use, excessive alcohol consumption, and betel quid chewing. OSCC is 2–3 times more prevalent in men than it is in women, and its incidence is the highest in people who are older than 50 years of age. Genetic predisposition also plays an important role in the development of OSCC [2,3].

Oral carcinogenesis is a highly complex, multifactorial, and multistep process that can begin as hyperplasia/hyperkeratosis and can evolve to epithelial dysplasia, carcinoma in situ, and OSCC [4]. Most OSCC are preceded by asymptomatic clinical lesions that are referred to as oral potentially malignant disorders (OPMDs), which include leukoplakia, erythroplakia, reverse smoker’s palate, erosive lichen planus, oral submucous fibrosis, lupus erythematosus, and actinic keratosis [5,6]. The clinical presentations of OPMDs can be further diagnosed as hyperplasia/ hyperkeratosis (HK), oral epithelial dysplasia (OED), or OSCC via histopathological evaluation. Epithelial HK are a benign overgrowth of cells in the oral epithelium. They may represent the initial stage of cancer development. OED is defined as a precancerous lesion in the oral epithelial region where cells exhibit atypia up to a certain level of the epithelium. The diagnosis and grading of OED are mainly based on the combination of architectural changes and the appearance of specific histological features [7]. An OED can be graded as mild, moderate, or severe based on the WHO’s three-tier classification system. It has been estimated that 7–50% of severe, 3–30% of moderate, and <5% of mild OED lesions can transform into OSCC [8,9,10].

The gold standard WHO 2017 three-tier grading system for OED has some limitations, including subjectivity, inter- and intra-observer variations, and limited capability in predicting the malignant transformation risk of OED in individual cases [11]. Suggestions to overcome these limitations include the use of clinical determinants and molecular markers to supplement the grading system [12]. However, no single clinical-pathological predicting factor or molecular biomarker has achieved the clinical criteria for that purpose [13]. Accurate risk assessment and the effective management of OPMD and OED play critical roles for improving oral cancer survival rates and prognosis. Therefore, there is a need for new biomarkers or modern techniques that can provide objective and accurate OPMD/OED risk stratification for early oral cancer detection and prevention.

One promising technique is Fourier transform infrared (FTIR) spectroscopy. FTIR spectroscopy is based on the vibrational energy state changes of molecules after absorbing infrared radiation at certain frequencies. The unique absorption pattern of a sample produces characteristic bands in its FTIR spectrum. The FTIR spectrum for a biological sample provides a biochemical profile of proteins, nucleic acids, lipids, and carbohydrates in the sample, called “biomolecular fingerprinting” [14]. Not only can FTIR spectroscopy measure the relative quantity of a certain biomolecule, but it is also sensitive enough to probe subtle changes in molecular structure and microenvironment, such as the secondary structure of proteins, the mutation of nucleic acids, and the peroxidation of phospholipids [15,16,17,18,19]. It has been shown that FTIR spectroscopy can detect bimolecular changes that are associated with carcinogenesis much earlier than the appearance of morphological abnormalities, supporting its promising role in early cancer detection [20,21,22]. In FTIR imaging, each individual pixel comprises a full FTIR spectrum, and both the spectral and spatial information of the sample is integrated into a three-dimensional hyperspectral data cube [23]. Since the middle of the 20th century, FTIR spectroscopy and imaging techniques have been studied as label-free, non-invasive, highly sensitive, and specific analytical tools for the detection and characterization of malignancies in a wide variety of tissues, including skin, brain, breast, colon, cervix, lung, stomach, ovary, prostate, leukemia, lymphoma, and squamous epithelium [22,24,25].

In the field of oral disease research, FTIR spectroscopy and imaging techniques have been used to investigate oral cancer and precancer using a variety of biological samples, including oral tissues, exfoliated oral cells, biofluids (e.g., serum, plasma, saliva, sputum), and extracellular vesicles. Those studies provide early evidence for the usefulness of FTIR in oral cancer characterization and the differentiation of cancerous samples from noncancerous ones [26]. However, the number of published studies so far is still relatively small in this area, and more research is needed to better understand the promise of FTIR in oral cancer detection and the potential for clinical translation.

In the current study, we report an accurate discrimination of OSCC biopsy samples from HK samples using transmission FTIR imaging technique together with machine learning algorithms. Particularly, we introduce a novel classification strategy for OED samples based on their FTIR spectral similarities to either HK or OSCC samples for the first time. This novel classification strategy is easy to implement computationally and may provide a potential risk stratification solution to the malignant progression assessment of OED. The specific objectives of the current study were: 1. to develop an effective and practical method of generating representative epithelial FTIR spectra from formalin-fixed paraffin-embedded (FFPE) biopsy samples; 2. to characterize HK, OED, and OSCC samples based on their representative spectra; 3. to build machine learning models for discriminating OSCC from HK samples; and 4. to develop a novel strategy for classifying OED samples for potential risk stratification applications.

## 2. Materials and Methods

**Figure 1:**
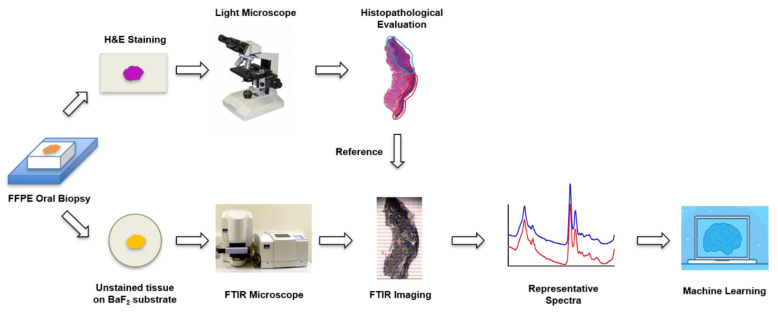
Flowchart of the experiment.

The overall flowchart of the experiment is illustrated in Figure 1.

### 2.1. Sample Preparation

The current study received approval from the Institutional Review Board of University of Missouri at Kansas City (UMKC) for the use of archived human oral tissues. Specifically, 34 FFPE archived oral biopsy samples were obtained from the Pathology Department of the UMKC School of Dentistry, including 12 HK samples, 11 moderate-to-severe OED samples, and 11 OSCC samples. The samples were cut into 5-µm sections using a manual microtome (Leica RM2125, RTS, Leica Biosystems Inc., Buffalo Grove, IL, USA). One section was placed on positively charged glass slides for hematoxylin and eosin (H&E) staining and histological evaluation. The H&E-stained sections were imaged with a light microscope (Keyence BZ-X810, Keyence Corporation, Osaka, Japan), and the digital images were sent to a pathologist, who subsequently annotated areas of interest (AOI) based on histopathological evaluation. The annotated H&E images were then used as references for FTIR imaging. An adjacent tissue section was placed on a BaF_2_ disc (REFLEX Analytical Corporation, Ridgewood, NJ, USA) for FTIR imaging. The tissue samples on the BaF_2_ discs were deparaffinized through immersion in histological grade xylene (CAS number 1330-20-7, Sigma-Aldrich, St. Louis, MO, USA) for 5 min × 3 times at room temperature. The deparaffinized samples were air-dried and stored in a vacuum desiccator.

### 2.2. FTIR Imaging

**Figure 2:**
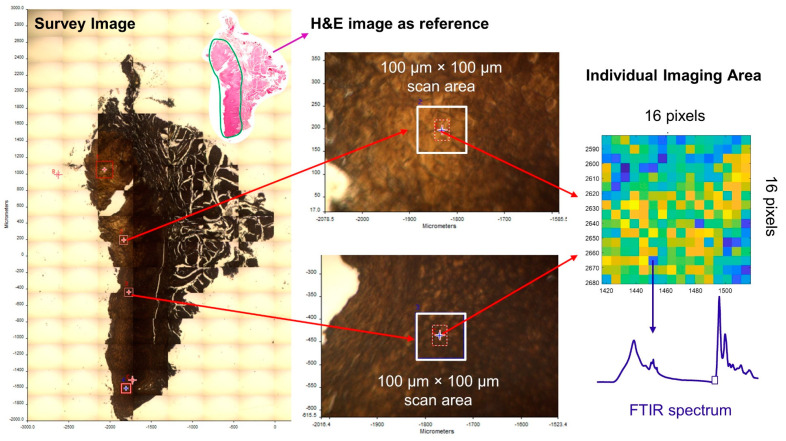
Illustration of FTIR imaging areas for a sample section.

FTIR images of tissue sections were acquired in transmission mode using a Perkin Elmer FTIR Spectrum Spotlight imaging system (Spectrum one, Spotlight 300, Perkin Elmer, Waltham, MA, USA). The Spotlight 300 imaging system features a dual-mode detector with a 1 ×16 narrow band mercury cadmium telluride (MCT) array and 100 µm medium band MCT single point detector operating at liquid nitrogen temperature. The following parameters were used for FTIR imaging: spectral resolution of 4 cm^−1^, spectral range of 4000–950 cm^−1^, pixel resolution of 6.25 µm, and co-adding spectra of 16 per pixel. Specifically, an overall survey image was first generated using the built-in light microscope in the FTIR Spotlight system for the sample section. Then, a series of 100 µm × 100 µm (16 × 16 pixels) imaging areas were defined in the survey image in reference to the diagnostic AOI in the corresponding digital H&E image. The diagnostic AOI was the sample region(s) that were used for pathological diagnosis. For example, if an OSCC sample consisted of hyperkeratotic region(s), dysplastic region(s), and OSCC region(s), only the OSCC region(s) were used as the diagnostic AOI. The imaging areas that were chosen were primarily in the epithelial regions for the HK and OED samples and in the invasive regions for the OSCC samples. Areas with poor tissue structural integrity were avoided to ensure high quality of spectra. The number of the imaging areas was mainly determined by the size and quality of each AOI. Right before the scan, a background spectrum was collected outside the sample area from the clean BaF_2_ substrate to be subtracted from the single beam spectra for background correction. The imaging area size of 100 µm × 100 µm was chosen to ensure that there was enough tissue/cell content to obtain a local representative spectrum while limiting the acquisition time to ensure a valid background correction. FTIR image acquisition was performed using the Spectrum IMAGE software by Perkin Elmer. Figure 2 illustrates the FTIR imaging areas as described above.

### 2.3. Data Analysis

FTIR hyperspectral images are high-dimensional data containing thousands of variables (spatial coordinates and wavenumbers) for many objects (samples). An FTIR hyperspectral dataset holds an enormous amount of biochemical information and requires appropriate multivariate analyses to identify patterns and trends as well as to build classification models.

#### 2.3.1. Spectral Preprocessing

All of the FTIR spectra were first preprocessed to remove or reduce biochemically irrelevant signal contributions from physical, macro-structural, and environmental factors. Spectral preprocessing can improve the accuracy of subsequent multivariate data analyses toward building better classification models. The data analysis was performed using the Eigenvector PLS_Toolbox software (Eigenvector research incorporated Inc., Manson, WA, USA) in MATLAB (R2020b, MathWorks, Inc., Natick, MA, USA). Specifically, the original hyperspectral image datasets were subject to the following general preprocessing: 1, transmission to absorbance conversion (A = log(1/T)); 2, selection of fingerprint region (1800–950 cm^−^^1^); 3, Savitzky–Golay smoothing; 4, EMSC (extended multiplicative signal correction) for light-scattering; 5, automated weighted least squares (AWLS) baseline correction; and 6, vector normalization. The general preprocessing allowed the spectra to stay in their non-derivative form for easy observation and interpretation. Secondary derivative spectral differentiation (7-point window size) was used as an additional preprocessing step during model building.

#### 2.3.2. Unsupervised Exploratory Analysis Using PCA and HCA

After signal preprocessing, unsupervised exploratory analyses were used to identify cluster patterns and data trends and to help understand the nature of the samples, including outliers and experimental errors. Principal component analysis (PCA) is the most widely used unsupervised multivariate exploratory analysis method for reducing the complexity of a spectral dataset by linearly transforming the original coordinate system into a new coordinate system defined by the principal components (PCs) that best explains the variance in the dataset. The PCs are orthogonal to each other and are generated in a decreasing order of explained variance. PCA decomposition uses the following form:

$$X\;=\;tp^{T}\;+\;E$$

where *X* represents the preprocessed spectra data, *t* represents the PCA scores, p represents the loadings, and *E* represents the residuals. All of the components are in matrix format, and *p^T^* represents the transpose of the loading matrix *p* [27]. The PCA scores represent the variance in the samples and are used to detect clustering patterns related to biochemical similarities or dissimilarities among the samples. The PCA loadings represent the variance in the wavenumbers and are used to identify important spectral variables for the pattern observed in the score distribution [28]. The PCA loadings are often used for identifying spectral biomarkers that distinguish samples in different biological or pathological classes.

An observation of each individual image dataset revealed some outlier spectra, which were removed using PCA. Specifically, a reduced Hotelling’s T^2^ versus Q residuals scattering plot chart was generated by PCA. The x-axis (reduced Hotelling’s T^2^) is the sum of the normalized squares scores, which is the distance from the multivariate mean to the sample projection onto the PCA PCs space. The y-axis (reduced Q residuals) is the sum of squares of each sample in the PCA error matrix. The pixel spectra with high value in Hotelling’s T^2^ or Q residual or both were investigated and were subsequently removed from the dataset.

Some FTIR imaging areas contained both epithelial and nonepithelial (e.g., stroma) tissues. Unsupervised hierarchical cluster analysis (HCA) was used to separate epithelial spectra from other types of spectra. Due to the distinct spectral features of different tissue types, HCA was able to separate them at the highest or the next highest hierarchical levels. The pixel spectra corresponding to the epithelial tissue were selected and averaged to generate a representative epithelial FTIR spectrum for each imaging area (referred to as “representative spectra” later).

Multiple representative spectra were generated for each sample and were visually examined for quality check. Intra-sample variations of representative spectra were observed for some samples. To address this issue, two high-quality representative spectra from the diagnostic AOI of each sample were selected to reflect the intra-sample heterogeneity. As a result, a total of 68 representative spectra were selected from 34 samples and were consolidated into one combined dataset for further exploratory and discriminant analyses. Each representative spectrum in the combined dataset was labelled with a class ID according to its histopathological diagnosis (H for HK, D for dysplasia, and C for OSCC). The class average spectra for the three classes were compared for the visual identification of spectral differences. Additional exploratory analyses using unsupervised PCA and HCA were performed on all 68 representative spectra in the combined dataset to identify patterns and trends.

#### 2.3.3. Supervised Discrimination between HK and OSCC Samples

Following exploratory analyses, discriminant machine learning models were built using three different supervised algorithms: partial least squares discriminant analysis (PLSDA), support vector machines discriminant analysis (SVMDA), and extreme gradient boosting discriminant analysis (XGBDA). PLSDA is a feature extraction and classification algorithm that is widely used for spectral data analysis. It is adapted from the partial least square regression (PLSR) technique, which aims to build a linear regression model using a latent variable (LV) approach to find the multidimensional direction in the *X* space that explains the maximum multidimensional variance direction in the *Y* space. The underlying mathematical model of PLSR is:

$$X\;=\;tp^{T}\;+\;E\;$$

$$Y\;=\;uq^{T}\;+\;F$$

in which *X* and *Y* are the observable variable matrix and predicted variable matrix, respectively; t and u represent projected scores of *X* and *Y*; p and q represent orthogonal loading matrices for the projected *X* and *Y* scores; and *E* and *F* are the error terms. When the predicted variables *Y* are categorical, such as in the current study (e.g., HK and OSCC classes), it becomes a discriminant technique called PLSDA. The PLSDA model is applied to *X*, reducing the original observable variables to a small number of LVs, which are linear combinations of the original variables that attempt to explain the maximum covariance between *X* and *Y*. Then, a linear discriminant classifier is used for classifying the samples [29]. PLSDA is an effective and powerful method for spectral data classification. It works well when high dimensionality and high collinearity are present in small-sample data, such as in the case in the current study. However, its performance may be subject to degradation under complex conditions such as nonlinearity, class imbalance, and multiclass [30]. An SVM is a binary linear classifier with a non-linear step called the kernel transformation [31]. A kernel function can transform the input spectral space into a feature space by applying a non-linear mathematical transformation. Then, a linear decision boundary is fit between the closest samples to the border of each class (called support vectors) and is used for determining the class memberships of new samples. In the current study, the radial basis function (RBF) kernel was used in SVM modeling. SVMDA is an effective algorithm for high dimensional spaces, especially when the number of dimensions is greater than the number of samples. With its kernel function, it can handle some non-linearity in the data. However, it is more time consuming and more susceptible to overfitting compared to PLSDA. The third algorithm XGBDA is an implementation of gradient boosted decision trees, that produces a prediction model in the form of an ensemble of weak prediction models, typically decision trees [32]. It is used in Kaggle competition and has shown superior efficiency and high prediction accuracy. The XGBDA algorithm is a class of lifting algorithm composes of a series of base classifiers. The original dataset is divided into multiple sub-datasets, and each sub-dataset is randomly assigned to the base classifier for classification/prediction. The results from the weak base classifiers are combined based on a certain weight, generating a final result for the XGBDA [33]. The advantages of XGBDA include its ability to handle non-linear parameters better than PLSDA and SVM and its robustness to outliers. On the other hand, XGBDA has a tendency for overfitting, and its performance for spectral data analysis has not been widely tested. It would be interesting to compare it with the commonly used PLSDA and SVMDA in the current study.

#### 2.3.4. A novel Strategy for OED Classification

Based on the results from the exploratory analyses, a novel strategy was developed for discriminant analysis. Specifically, in the first phase, 46 representative spectra from 12 HK samples and 11 OSCC samples were used as training data to build the discriminant models. Due to the relatively small sample size, venetian blind (10-fold with 2 spectra from the same sample per blind) cross-validation was used for model performance optimization and evaluation. The model performance was evaluated using receiver operating characteristic curves (ROC curves), area under the curve (AUC), and sensitivity and specificity. A confusion matrix provides information for true positive (TP), false positive (FP), true negative (TN), and false negative (FN). Sensitivity is defined as the probability of achieving a positive test result in subjects with the disease and can be calculated by TP/(TP + FN). Specificity is defined as the probability of obtaining a negative test result in subjects without the disease and can be calculated by TN/(TN + FP) [34]. In the second phase, the performances of the three discriminant models were compared, and the best performing model was further used to classify the 22 representative spectra from the 11 OED samples.

## 3. Results

**Figure 3:**
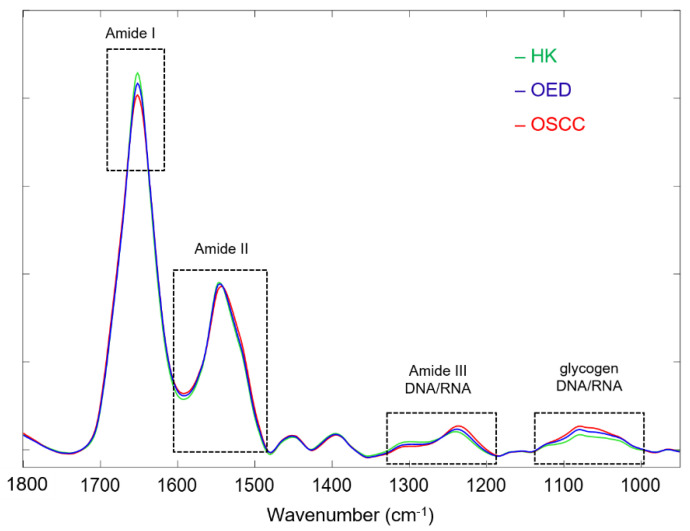
Three class-average spectra after general preprocessing for HK (green), OED (blue), and OSCC (red) samples, with visible spectral differences highlighted in dashed boxes.

Figure 3 compares the class-average spectra for the HK, OED, and OSCC samples. Specifically, the class average spectra were calculated from 24 representative spectra of HK samples (green), 22 representative spectra of OED samples (blue), and 22 representative spectra of OSCC samples (red), respectively. Based on visual observation, the spectral differences are mainly located in four spectral regions: the region around 1650 cm^−1^, the region of 1600–1500 cm^−1^, the region of 1350–1180 cm^−1^, and the region of 1160–950 cm^−1^. The 1650 cm^−1^ band is the amide I band of protein, which is mainly associated with the C=O stretching vibration in the peptide backbone structure [35]. The results show a descending band intensity in the order of HK > OED > OSCC for the amide I band. The 1600–1500 cm^−1^ region is the amide II band of the protein, which is mainly associated with the bending vibration of the N-H bond and the stretching vibration of the C-N bond in the peptide backbone [35]. The results show a descending band intensity at 1548 cm^−1^ in the order of HK > OED > OSCC and a red shift toward lower wavenumbers on the right shoulder of the amide II band for the OSCC and OED spectra (more shift for OSCC than OED). The spectral region of 1350–1180 cm^−1^ can be attributed to the amide III band of protein (1350–1250 cm^−1^), which is mainly from N-H bending and C-N stretching vibration, to the asymmetric vibration of –PO_2_^−^ (1240 cm^−1^), and to the deformational modes of the CH_3_/CH_2_ groups in phospholipid and nuclei acids [36,37]. The results show a descending band intensity at 1310 cm^−1^ in the order of HK > OED > OSCC and at 1240 cm^−1^ in the order of OSCC > OED > HK. The spectral region of 1160–950 cm^−1^ can be attributed to the stretching vibrations of the C–O/C–C groups in the carbohydrate (e.g., glycogen) (1154 and 1030 cm^−1^) and to the symmetric vibration of –PO_2_^−^ in the phospholipid and nucleic acids (1080 cm^−1^) [14,38]. The results show a descending band intensity in this region in the order of OSCC > OED > HK.

**Figure 4:**
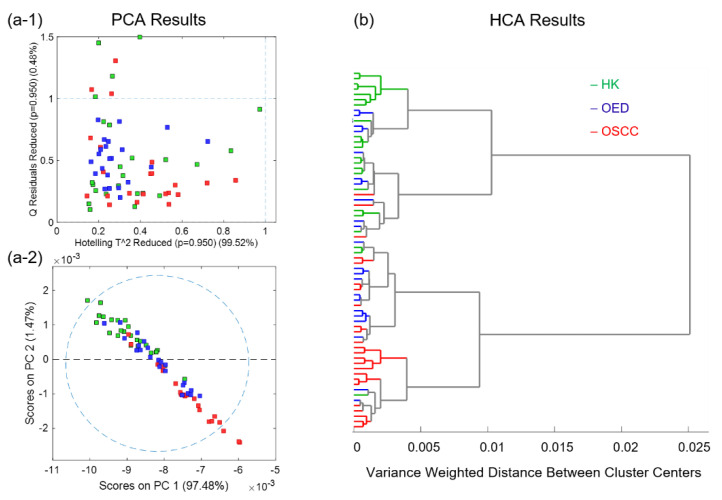
Unsupervised analysis results for all 68 representative spectra in the combined dataset: (**a**-**1**) Reduced Hotelling T^2^ versus Q residuals graph of principle component analysis (PCA), and (**a**-**2**) score plot for principal components (PC1 and PC2) of PCA; (**b**) dendrogram graph of hierarchical cluster analysis (HCA).

Figure 4 shows the exploratory analysis results for all 68 representative spectra. (a-1) shows the reduced Hotelling T^2^ versus Q residuals graph of PCA; (a-2) shows the score plot for PC1 and PC2; and (b) shows the HCA dendrogram graph. Both the PCA and HCA results showed good but not ideal separation between the HK and OSCC representative spectra. The OED representative spectra overlap with both the HK and OSCC representative spectra.

**Figure 5:**
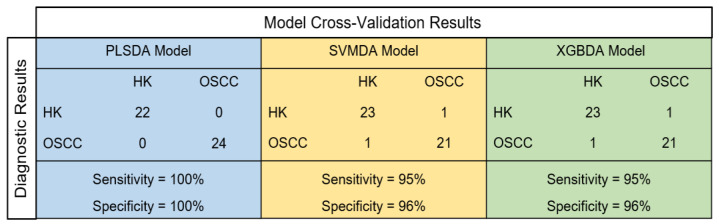
Cross-validation performances of three machine learning models for discriminating OSCC from HK samples. PLSDA—partial least square discriminant analysis, SVMDA—support vector machine discriminant analysis, and XGBDA—extreme gradient boosting discriminant analysis.

Figure 5 summarizes the cross-validation performances of the three machine learning models built in the current study (PLSDA, SVMDA, and XGBDA) for discriminating the OSCC from HK samples. The PLSDA model showed a 100% sensitivity and 100% specificity, while both the SVMDA and the XGBDA models showed 95% sensitivities and 96% specificities.

**Figure 6:**
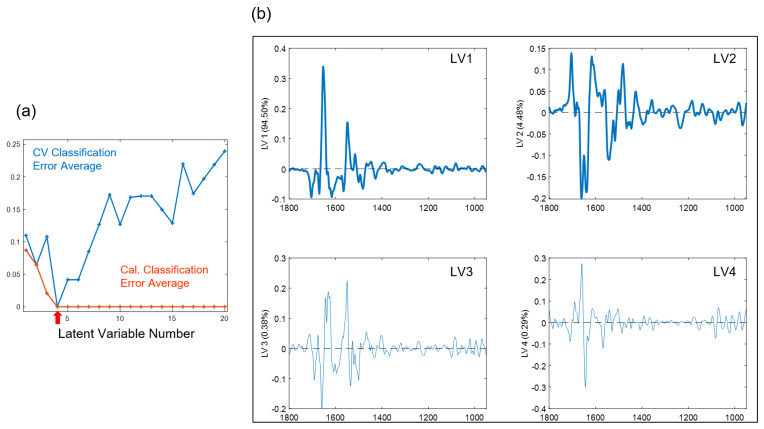
(**a**) Average calibration (Cal.) and cross-validation (CV) modeling errors versus the number of latent variables (LV) and (**b**) four chosen latent variable loadings (LV1–LV4) for the partial least square discriminant analysis (PLSDA) model.

Figure 6a shows the average modeling errors on the y-axis versus the number of latent variables (LV) on the x-axis for the PLSDA model. Both the average classification errors for the calibration (orange curve) and cross-validation (blue curve) were displayed. The optimal number of latent variables was chosen to be four to minimize both errors. Figure 6b shows the loadings of the four chosen LVs for the PLSDA model. The loadings of LV1, LV2, LV3, and LV4 explain 94.50%, 4.48%, 0.38%, and 0.29% of the spectral variations between the HK and OSCC samples, respectively. Particularly, the loading of LV1 shows several prominent bands at 1670 (−), 1654 (+), 1548 (+),1516 (+), 1482 (−), 1238 (+), 1082 (+), 1026 (+), and 966 (+) cm^−1^, and the loading of LV2 shows several prominent bands at 1704 (+), 1660 (−), 1640 (−), and 1482 (+), where “+” indicates positive bands and “−” indicates negative bands. Table 1 summarizes the 12 feature bands that were extracted from the top two loadings (LV1 and LV2) of the PLSDA model and their corresponding vibrational modes and biochemical assignment. Those bands are considered spectral biomarkers for discriminating OSCC from HK samples.

**Figure 7:**
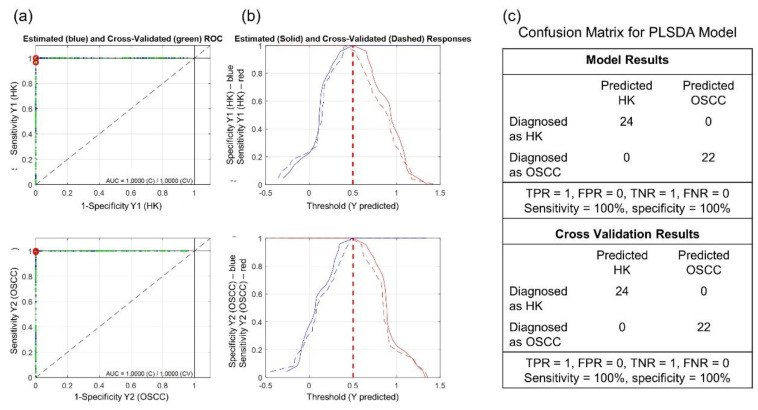
(**a**) Receiver operating characteristic (ROC) curves with area under the curve (AUC) values (AUC = 1); (**b**) sensitivity and specificity curves vs. threshold; and (**c**) confusion matrix for the partial least square discriminant analysis (PLSDA) model. TPR is true positive ratio, FPR is false positive ratio, TNR is true negative ratio, and FNR is false negative ratio.

Figure 7 shows (a) the ROC curves and AUC values, (b) the sensitivity and specificity curves, and (c) the confusion matrix for the PLSDA model. The AUC for calibration and cross-validation were both 1, and the sensitivity and specificity were both 100% at a threshold of 0.5 for the HK and OSCC samples. The results indicate a perfect discriminant model for the experimental HK and OSCC spectral data.

**Figure 8:**
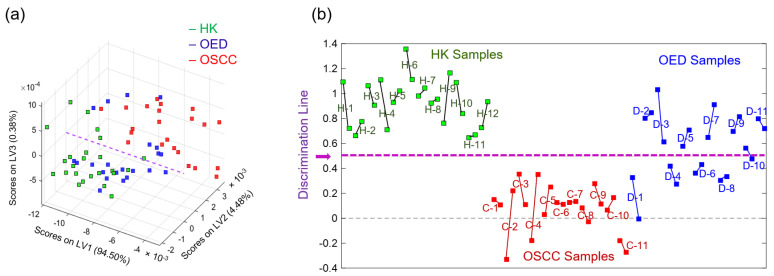
(**a**) Score plot for the top three latent variables (LV1–LV3) of the partial least square discriminant analysis (PLSDA) model; (**b**) visual illustration of the discrimination of all 34 samples by the PLSDA model (H for HK samples, C for OSCC samples, and D for OED samples).

Figure 8a shows the PLSDA model score plot for the top three latent variables (LV1–LV3) for all 68 representative spectra. Figure 8b visually summarizes the discrimination results for all 34 samples. The dashed purple line in the middle is the discrimination line of the PLSDA model. Two representative spectra connected with a solid line came from the same sample—a longer line suggests higher intra-sample spectral heterogeneity, while a shorter line suggests lower intra-sample spectral heterogeneity. The results show a complete discrimination between all of the HK samples and OSCC samples. For the OED samples, D-2, D-3, D-5, D-7, D-9, and D-11 were classified as “HK-grade”, indicating more spectral similarities between the six OED samples and the HK samples; D-1, D-4, D-6, and D-8 were classified as “OSCC-grade”, indicating more spectral similarities between the four OED samples and the OSCC samples. The two representative spectra of the sample D-10 were located in the vicinity of both sides of the discrimination line, indicating a borderline classification case.

## 4. Discussion

Despite cancer treatment advancements, oral cancer survival rates have not improved over the past several decades. The gold standard histopathological diagnosis of oral cancer and the grading of oral epithelial dysplasia have many limitations, including subjectivity, inconsistency, and inaccuracy. The lack of an effective screening program and the challenge of managing potentially malignant disorders and precancerous dysplasia lead to late diagnosis in >70% oral cancer patients, resulting in poor prognosis and survival rates. It has been shown that most oral cancer patients have pre-existing OPMDs, the majority of which are diagnosed as HK or OED [40]. OED is characterized by cytological and architectural alterations reflecting the loss of the normal maturation and stratification pattern of surface epithelium. Although these lesions have an increased statistical risk of progressing to malignancy, it is very difficult to predict the outcomes for individual patients with current histopathological diagnostic methods [12]. For example, even in the case of severe OED, the malignant transformation rate varies considerably from 3% to 50% [11]. According to Tilakaratne et al., a successful OED grading system should be (i) clinically relevant in terms of stratifying the cases for appropriate management plans, (ii) reproducible, minimizing intra- and inter-examiner variability, and (iii) biologically significant by identifying the lesions that are likely to undergo malignant transformation [41]. FTIR spectroscopy and imaging techniques show great promise to meet these criteria and to address the unmet medical needs for objective and accurate OED risk stratification.

A number of studies have applied FTIR techniques to investigate biochemical differences between normal and malignant oral tissues in the past two decades. Schultz et al. observed that poorly differentiated OSCC cells produced a relatively homogeneous and clearly abnormal cell biochemistry, whereas well-differentiated epithelial cells presented a very heterogeneous distribution of cellular components. The authors suggested that the FTIR analysis of cell components can be used to distinguish cancerous tissues from normal epithelial structures [42,43]. Fukuyama et al. observed FTIR spectral differences between normal oral mucosa and OSCC, including bands related to keratin, collagen, phosphate of nucleic acids, and membrane phospholipids [44]. A few studies on normal, pre-cancerous, and cancerous tissues of oral cavity have been conducted by one group from Università Politecnica delle Marche in Ancona, Italy, using reflectance FTIR mapping of thin tissue sections on a steel support. Distinct FTIR chemical maps of vibrational bands at 970 cm^−1^ (DNA), 1026 cm^−1^ (collagen), 1550 cm^−1^ (proteins), and 1735 cm^−1^ (lipids) were observed between normal and pathological oral tissues. The authors reported that the proliferating and regressive states of the tumors can be identified via the presence of a high content of DNA or collagen, respectively [45]. Pallua et al. investigated microarrays of OSCC tissues using FTIR imaging with unsupervised methods including HCA and KMC (k-means cluster) and showed that intra-operative and surgical samples of the oral cavity can be characterized by FTIR microscopic imaging [46]. Bruni et al. reported increased DNA, lipid, and collagen levels in OSCC samples [45]. However, their attribution of the 1026 cm^−1^ band to collagen is debatable, as many other studies assigned the same band to glycogen instead [47,48,49,50]. Sabbatini et al. performed vibrational analyses of both epithelial and connective tissues of OSCC at various malignancy grades (G1–G3) and identified potential spectral markers for oral carcinogenesis, including the increase of free glycogen levels, structural alterations in nucleic acids, and a higher amount of RNA, which suggests an increase of the cellular transcriptional activity [51]. Banerjee et al. investigated FTIR-based spectral biomarkers towards the optimal differentiation of oral leukoplakia and cancer using a different sample preparation method. Specifically, deparaffinized FFPE tissue sections were treated as powder to prepare KBr pellets from which transmission spectra were acquired. The spectra represented a mixture of all of the components of the sample, including epithelial and connective tissues. They identified more than 20 spectral biomarkers using difference between mean spectra, forward feature selection, and Mann–Whitney U test techniques. The identified biomarkers were assigned to amide I, amide II, lipid, keratin, glycogen, DNA/RNA, etc. [48]. Naurecka et al. used the FTIR-ATR technique to study normal, leukoplakia, and cancerous oral tissues and reported spectral differences at amide I at 1650 cm^−1^, amide II at 1535 cm^−1^, nuclei acids at 1238 cm^−1^, and glycogen at 1024–1030 cm^−1^ [49].

In the current study, the FTIR imaging technique combined with multivariate analyses were used to evaluate three classes of oral biopsy samples (HK, OED, and OSCC). Since whole sample FTIR imaging is very time consuming, a practical imaging method was developed to acquire representative spectra from the sample in a short time. Specifically, a series of 100 µm × 100 µm imaging areas (16 by 16 pixels) were defined in the AOI of the sample in reference to the corresponding H&E image. The area size of 100 µm × 100 µm was chosen so that each area contained enough tissue/cells to generate a local representative spectrum, while the acquisition time for each area was reasonably short (5–6 min) to ensure valid background correction. Representative spectra from multiple imaging areas in a sample were reviewed to understand the tissue heterogeneity of the specific sample. Different degrees of heterogeneity were observed for different samples. The consideration of intra-sample heterogeneity is particularly important for accurate model training when a relatively small number of training samples are used since a large number of training samples may contain high enough inter-sample heterogeneity to compensate for intra-sample heterogeneity. On the other hand, too many representative spectra from the same sample may cause a data redundancy problem, especially for samples with a low degree of intra-sample heterogeneity. As a result, a simple strategy was used to select the two representative spectra that best reflected the intra-sample heterogeneity of each sample for further analysis.

A series of spectral preprocessing was applied to the raw data to remove unwanted signal contributions, such as those from sample thickness variations and light scattering, and to prepare the data for optimal performance in later steps. The choice and quality of spectral preprocessing play an important role in the performance of multivariate analysis and classification. Even the order of the preprocessing steps can affect the analysis results. In the current study, different preprocessing steps and parameters were tried and the optimal procedure was decided as the following: the fingerprint region of 1800–950 cm^−1^ was selected due to its association with major biochemicals in tissues; second-order Savitzky–Golay smoothing was used to remove random noise (e.g., from instrument) while preserving useful biochemical spectral information; the EMSC algorithm was used to correct for resonant Mie scattering while maintaining the original spectral shape and scale; AWLS baseline correction was applied to remove background absorption interference, and vector normalization was then employed to correct for sample thickness variations. The above steps comprised the general preprocessing, which preserved the original shape of the spectra for easy visual comparison and interpretation. A comparison of the three class average spectra for the HK, OED, and OSCC samples (Figure 3) reveals an overall reduction in proteins and an increase in the nucleic acids as the oral tissues progress from HK to OED and further to OSCC. The amide II band exhibits a red shift for OED and OSCC samples, indicating some secondary structural changes in the collagen of the pathological tissues. Those findings are in good agreement with the literature that normal tissue spectra are characterized by higher protein contents, whereas more DNA and lipid signals are exhibited by malignant tissues [52]. In the current study, the typical lipid band between 1750–1700 cm^−1^ disappeared in all of the spectra, which is most likely due to the deparaffinization procedure which removed free lipids in the tissues as a side effect of removing the paraffin of the FFPE samples.

Exploratory analyses using PCA and HCA verified the overall quality and showed the trend of all 68 representative spectra. The reduced Hotelling T^2^ versus Q residuals graph of PCA revealed no outliers among the data. The PCA and HCA results revealed a good but not ideal separation between the HK and OSCC spectra. The OED spectra were shown to overlap with both the HK and OSCC spectra. Exploratory analyses using unsupervised methods such as PCA and HCA serve well for quality inspection, patten observation, and trend discovery, but they usually cannot provide optimal classification for complicated pathological samples. Supervised methods are required for that purpose.

Based on the patterns revealed in the exploratory analyses, the OED spectra do not seem to separate well from either the HK spectra or the OSCC spectra. Instead, some OED spectra exhibit similarities to the HK spectra, while others exhibit similarities to the OSCC spectra. As a result, a novel strategy was developed to build an optimal discriminant model using the representative spectra of the HK and OSCC samples (first phase), which was subsequently used to classify the representative spectra of the OED samples (second phase).

Additional spectral preprocessing was applied to the representative spectra to optimize the discrimination model. First and second derivatives are commonly used preprocessing steps to highlight smaller spectral differences, which can be critical for finding the discriminative spectral features for complex biological samples. In the current study, the modeling results for non-derivative, first derivative, and second derivative representative spectra were compared. It was found that the second derivative preprocessing produced the best performance for the discrimination models. Therefore, it was used for final model building and validation.

In the first phase, three supervised discrimination models (PLSDA, SVMDA, and XGBDA) were trained and cross-validated using 24 HK spectra and 22 OSCC spectra. The results show better discrimination performance for the PLSDA model (100% sensitivity and 100% specificity) than the SVMDA and XGBDA models (95% sensitivity and 96% specificity). A total of 12 prominent bands were extracted from the top two latent variable loadings (LV1 and LV2) of the PLSDA model as discriminative spectral biomarkers in differentiating the OSCC from the HK samples (feature extraction/selection), as summarized in Table 1. The discriminative bands mainly came from proteins (amide I/II), nucleic acids (–PO_2_^−^), and carbohydrates (glucose and glycogen, etc.).

In the second phase, the optimal PLSDA model was used to classify the 22 representative spectra from 11OED samples. The classification results show that 6 OED samples were classified as “HK-grade”, indicating their spectral similarities to the HK samples, and 4 OED samples were classified as “OSCC-grade”, indicating their spectral similarities to the OSCC samples. One OED sample was classified as “borderline case” because its two representative spectra were in proximity to and on both sides of the discrimination line. The 11 OED samples looked very similar in their morphological appearance and were all pathologically diagnosed as having a moderate-to-severe grade of dysplasia. However, The PLSDA model was able to classify them based on their FTIR spectral information as being biochemically similar either to the HK samples or to the OSCC samples. The results suggest that those morphologically similar tissue samples exhibit different biochemical profiles that can be detected using the FTIR-machine learning approach. The novel strategy developed in the current study provides a potential risk stratification method for OED.

A total of 68 representative FTIR spectra from 34 samples was used in the current pilot study. Future studies with a larger sample size are needed to further improve and validate the OSCC discrimination model. Moreover, archived OED cases with known OSCC transformation outcomes are needed to validate this novel strategy in its efficacy of predicting the malignant transformation risks of OED cases.

In the current study, a traditional Perkin Elmer FTIR spectrometer and imaging system (Spectrum one, Spotlight 300, Perkin Elmer, Waltham, MA, USA) with the spectral resolution of 4 cm^−1^ and spatial resolution of 6.25 µm was used. The current FTIR image acquisition and spectral preprocessing protocols generated good quality spectra in a reasonable time frame. Further improvements in spatial resolution and spectral quality can be achieved with added lenses, high-resolution infrared microscope optics, computational algorithms, and quantum cascade laser imaging systems, which offer advantages over traditional FTIR systems with respect to the speed of acquisition and field of view [53].

## 5. Conclusions

In summary, within the limitations of the study, our results show that an FTIR-machine learning approach can discriminant OSCC from HK oral biopsy samples with high accuracy. The novel OED classification strategy developed in the current study could potentially provide an objective risk stratification tool for OED or OPMDs and could therefore facilitate the early detection of oral cancer. Tissue sections from FFPE samples are routinely used for histopathological evaluation in cancer clinics. The use of the same tissue sections (unstained) for FTIR imaging can be easily integrated into existing diagnostic procedures. The integration of FTIR imaging techniques in existing histopathological diagnostic process provides valuable biochemical evaluation in addition to morphological evaluation and can assist pathologists in making a more accurate risk assessment for OPMDs/OED and for the earlier detection for OSCC.

## Figures and Tables

**Table 1:** Spectral biomarkers identified using feature selection of the PLSDA model for discriminating OSCC from HK samples and their corresponding vibrational modes and biochemical assignments [14,39].

Wavenumber (cm^−1^)	Vibrational Modes and Biochemical Assignments
1704	Ester carbonyl C=O stretching, fatty acid esters, lipids
1670	Amide I, secondary structure of proteins
1660	Amide I, secondary structure of proteins
1654	C=O stretching of amide I, secondary structure of proteins,
1640	Amide I, secondary structure of proteins
1548	C-N and CN-H stretching of amide II, secondary structure of proteins
1516	Amide II, secondary structure of proteins
1482	deformation vibrations of –CH_3_, lipid
1238	Asymmetric phosphodiester stretching ν_as_ (–PO_2_^−^), lipid, nuclei acid, amide III (C-N stretching, N-H bending) proteins
1082	Symmetric phosphodiester stretching ν_s_ (–PO_2_^−^), protein phosphorylation, phospholipids, collagen, DNA
1026	Vibrational frequency of -CH_2_OH groups of carbohydrates (e.g., glucose, glycogen, etc.) C-O stretching, C-O stretching coupled with C-O bending of the C-OH groups of carbohydrates
966	C-O stretching of the phosphodiester, deoxyribose, C-C of DNA

## Data Availability

The data presented in this study are available on request.
